# Editorial: Fundamentals of 21st Century Neuroscience

**DOI:** 10.3389/fnmol.2021.835419

**Published:** 2022-01-27

**Authors:** Elsa M. Pellet, Annechien S. Helsdingen, Leonor M. Teles Grilo Ruivo

**Affiliations:** ^1^Center for Digital Education, Ecole Polytechnique Fédérale de Lausanne, Lausanne, Switzerland; ^2^Instituto Gulbenkian de Ciência, Oeiras, Portugal

**Keywords:** neurogenetics, neuroimmunity, neuroproteomics, synaptic plasticity, neural circuits, cognition, behavior, multi-scale neuroscience

Neuroscience needs a multidisciplinary approach and inter-institutional collaboration. To fully understand brain functioning and neurological disorders, there is a need to integrate knowledge of experimental and theoretical approaches at different levels and from different perspectives.

This Research Topic, developed in parallel with the design and production of online courses on the fundamentals of neuroscience (Neuroscience reconstructed, a MOOC developed by the EPFL), aims to provide an articulate, multi-scale view of brain function by incorporating knowledge ranging from the level of genes and molecules, to networks, to cognitive and behavioral neuroscience in the form of mini-reviews. In both the courses and this Research Topic, we wish to provide a basic understanding of the processes and structures and how we study these using the most promising new technologies.

The multi-scale aspect is reflected in all the mini-reviews in this Research Topic: several studies present a review of studies at the genetic level that help the understanding of constructs from a higher levels of analysis, such as evolution (Miller et al.), development (Aberle; Sullivan) and neurological disease (Coda andGräff; Tielking et al.). Similarly, mini-reviews focus on studies at the cellular level and how those can shed light on mechanisms related to activity in neuronal circuits (De-Miguel et al.) while also helping us understand higher-level mechanisms such as learning and memory (Borroto-Escuela and Fuxe). At an even higher level of analysis—circuits, systems, cognition and behavior—we find reviews that start from circuits and provide an overview of their role in addiction (Lipton et al.), or start from addiction and provide an overview of the role of different brain regions and neurotransmitter transporters in shaping such condition ( Wang et al.).

We have also included reviews that focus particularly on how new technologies and methods have provided opportunities for multi-level analysis and improved our understanding of brain function. All the way from the cellular level, where Magliaro et al. review tools available to study how neuron morphology relates to brain function, to the cognitive level, showcasing a review from George and Sunny on how Bayesian models can support our understanding and how modularity at the system level impacts cognition and function.

Looking at the breadth of reviews detailed below, the explanatory power of integrative, multi-level analysis becomes evident when attempting to understand complex phenomena that develop over disparate levels of neural organization.

## Interplay of Genetics and …

### Evolution, Structure, and Function

Miller et al. review the role of phylogenetics in understanding the relationship between structure and function in neuroscience and the disadvantages of studying few species amenable to transgenic manipulations in neuropharmacology, neural network architecture and connectome studies. They highlight how next-generation molecular tools such as CRISPR/Cas9 and viral approaches will allow the study of more diverse taxa and how comparative approaches could provide a better understanding of neural function.

### Anatomy, Cognition

Shah et al. present an overview of traumatic brain injury (TBI) mammalian models. They summarize the varied physical, behavioral and cognitive effects of TBI and discuss the advantages of different model organisms in TBI research with a focus on Drosophila. They describe the different experimental models used to generate TBI and review the findings about transcriptional changes induced by TBI in different model animals. Finally, they discuss the importance of animal models and experimental models as well as genetic studies for biomarker discovery and TBI therapy.

### Neurological Disorders

Coda and Gräff provide an overview of how genes, epigenetic mechanisms and the environment interact, and discuss the classical nature vs. nurture question under the light of recent findings about the nervous system's physiology. They summarize the established principles of gene regulation in the brain and review how integrated omics-based approaches have helped the understanding of neurological and neuropsychiatric disorders. Finally, they discuss the challenges posed by such large multi-scaled and multi-modal datasets.

### Development, Circuits

Aberle sums up the process of axon guidance by attractants and discusses its similarities to cell migration. They analyze how localized gene expression drives axon growth and how disruption of this gene expression leads to abnormal branching patterns.

### Development, Connectivity

Sullivan discusses how molecular and genetic mechanisms generating neuronal identity influence connectivity in the Drosophila brain. They review the role of spatial and temporal genes during neurogenesis in Drosophila and how these genes, together with Notch signaling, influence connectivity. Finally, they highlight the advantages of Drosophila as a simple model organism.

### Signaling, Immune System

Tielking et al. provide a summary of the current knowledge about extracellular RNA regulation, diversity, quantification and function, outlining what is known about the role of exRNAs in signaling to the immune system and the vasculature. They discuss the role of extracellular RNA in several central nervous system pathologies, including different CNS tumors, strokes and multiple sclerosis, as well as the potential treatment and diagnostic strategies based on extracellular RNAs.

## Interplay of Cellular Biology and …

### Signaling, Proteomics

Borroto-Escuela and Fuxe discuss how the complexity of G-protein-coupled receptor (GPCR) interactions can lead to molecular signal integration in the nervous system. They sum up the history of findings about GPCRs, their allosteric interactions and consequent effects on signaling mechanisms. They present the different classes of GPCRs heteromers as well as their biological functions and highlight the role of bioinformatics, mathematics, and structural models in understanding complex formation. They conclude with the role of GPCR heteromers as molecular substrates for learning and memory.

### Microbiota, Parkinson's

Yang et al. summarize the direct and indirect evidence linking the gut microbiota to Parkinson's disease (PD). After briefly introducing the role of the gut microbiota as the “second brain” in the gut microbiota-brain axis (GBA), they sum up the history of PD and its effects on the gut. They go on to discuss what is known about α-synuclein accumulation in the enteric nervous system, the role of entero-endocrine cells in connecting the gut and the brain and intestinal permeability. Finally, they debate the function of the GBA in PD pathology and treatment.

### Circuits

De-Miguel et al. deals with a non-synaptic form of communication, originating in neurons in response to action potentials or long depolarizations, that affects neuronal circuits, glia and vasculature. They review the historical evidence and cellular basis for electrophysiological activity—triggering exocytosis of signaling molecules from neurons outside of the synaptic cleft. Finally, they discuss the role of this form of communication in neuronal circuit activity modulation and in information integration.

### Plasticity, Proteomics

Jackson et al. focus on the role of synaptic loss in Alzheimer's disease (AD). They briefly review the concept of synaptic plasticity and the concept of AD as a synaptopathy before presenting the findings linking Amyloid and Tau to synaptic loss. Finally, they discuss the existing caveats in translational research in the field and the potential therapeutic applications targeting synaptopathy in AD.

### Adult Neurogenesis, Cellular Markers

La Rosa et al. review the history of adult neurogenesis research and highlight the large differences between species. They explain the concepts of niches as microenvironments allowing the regulation of neural stem cell maintenance and differentiation and the need for good species-specific markers that would allow reliable differentiation between stem cells and immature neurons. They discuss the importance of new tools, new markers, and comparative studies for translational purposes.

### Methodology—Cell Morphology, Brain Function

Magliaro et al. review the tools available to study how neuron morphology and connectivity relate to brain function and the importance of a complete digital map of the mammalian brain. They present the existing algorithms for neuron segmentation and the methods for tissue clarification. Finally, they discuss new methods for acquiring images at sub-cellular scales and the need for corresponding robust algorithms to identify neurons and their substructures.

## Interplay of Circuits and …

### Behavior

Lipton et al. review the neural circuits implicated in habits, compulsions and addictions. They explain what these three behaviors are and what they have in common, which neural circuits are involved in the automation of behavior and the paradigms used to model them. Focusing on the dorsolateral striatum, they discuss the role of different cell types and microcircuits in behavioral automaticity and the molecular and synaptic changes involved.

### Electrophysiology, Behavior

Augusto and Gambino explore how NMDA spike generation in pyramidal neurons impacts an animal's behavior according to *in vivo* studies. After presenting the biological basis of NMDA spikes, they review the importance of spatial clustering of NMDA spikes and their role in synaptic plasticity. Finally, they discuss the evidence for the ability of NMDA spikes to impact the detection of stimuli, memory, and skill learning.

## Interplay of the Immune System, the Nervous System and the Vasculature

Geyer et al. provide background information on the blood-brain barrier, the CNS as a compartmentalized immune-privileged site, bacterial infections of the CNS and the role of astrocytes and microglia as immune-effector cells. Specifically, they review recent findings about how astrocytes recognize pathogens, their role in neuro-inflammation and neuro-protection, and how they interact with T cells. They argue that a better understanding of astrocyte diversity can help develop better treatments against bacterial CNS infections.

Villabona-Rueda et al. explain the evolving concepts on the blood brain barrier (BBB) and the neurovascular unit (NVU). They present the history of the discovery of a barrier between the CNS and peripheral circulation and how this barrier is influenced by the neural environment. They highlight the differences in NVU composition across different brain regions and the importance of this vascular heterogeneity for brain function in health and disease.

Brown et al. review the functions of pericytes, multi-functional cells that are part of the NVU, in brain function and disease. After a short history of pericyte research, they sum up the known functions of pericytes in cerebral blood flow regulation, vascular development and maintenance as well as neuro-immunity. They review recent findings about the role of pericytes in neurological diseases and discuss the future therapeutic implications of new methods to characterize and classify pericyte subtypes.

## Interplay of Cognition and …

### Anatomy, Circuits

Perry and Mitchell discuss the role of thalamic nuclei in memory. They present the well-established role of the thalamus in memory formation and the circuits that the different nuclei are part of. They highlight the difference between first-order and higher-order nuclei and discuss the new evidence pointing to single subnuclei being composed of separate entities with different functions, some of them of a higher-order than passive relays.

### Anatomy

Wang studies the effect of Abacus training on cognitive functions. Wang reviews the effect of abacus-based mental calculation (AMC) on different cognitive processes such as mathematics, working memory, and fluid intelligence, and the functional and structural plasticity associated with it. Finally, they outline the caveats of existing AMC studies remaining to be addressed in future research.

### Methodology—Bayesian Brain Models

George and Sunny focus on the relationship between the modularity assumption in cognitive science and bayesian brain (BB) models. They start by reviewing the history behind the modularity principle and introduce the concepts of penetrability and top-down effects. They then discuss the implications of different BB models for modularity and how model predictions can be corroborated by empirical findings. Finally, they compare two theoretical frameworks to describe the perceptual and motor system and their interactions.

## Interplay of Behavior and …

### Proteomics

Wang et al. review the role of glutamate transporters in drug addiction. After outlining the main brain regions involved in addiction and giving an overview of glutamate transporters distribution, they present evidence for the role of glutamate transporters in addiction in different regions of the brain and discuss the potential of these transporters as therapeutic targets for treating addiction.

### Body and Brain

Xu et al. focus on human decision making. They introduce the somatic marker hypothesis (SMH), which proposes that emotions guide decision making; the theory of ecological rationality, which stresses the role of the environment in decision making; embodied emotions, which implies that emotions are defined by the body; and neuroeconomics, which links economics, psychology and neuroscience. They outline how SMH relates to ecological rationality, embodied emotion and neuroeconomics and discuss how SMH bridges all three disciplines.

### Methodology—Behavior, Metabolism

Ren et al. explore the use of near-infrared spectroscopy (NIRS) to monitor cerebral hemodynamics in sleep studies. They begin with an introduction to the physical principles of NIRS and its potential applications. They then introduce typical cerebral hemodynamics measured with NIRS during normal sleep stages and sleep-disordered breathing. They discuss how NIRS results relate to other physiological factors as well as the underlying biological mechanisms. They outline the main differences in experimental design and signal processing and end with an overview of future perspectives.

## Author Contributions

EMP wrote the manuscript. ASH and LMT designed the Research Topic and reviewed the manuscript. All authors contributed to the article and approved the submitted version.

## Conflict of Interest

The authors declare that the research was conducted in the absence of any commercial or financial relationships that could be construed as a potential conflict of interest.

## Publisher's Note

All claims expressed in this article are solely those of the authors and do not necessarily represent those of their affiliated organizations, or those of the publisher, the editors and the reviewers. Any product that may be evaluated in this article, or claim that may be made by its manufacturer, is not guaranteed or endorsed by the publisher.

